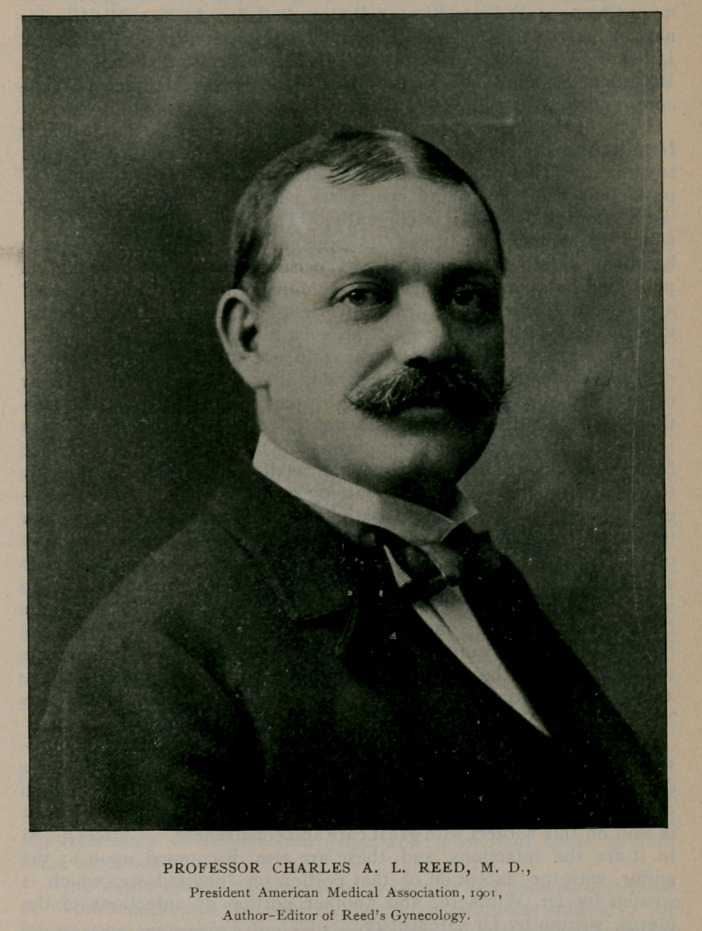# Book Reviews

**Published:** 1901-06

**Authors:** 


### Book Reviews.

A Text-Book of Gynecology. Edited by Charles A. L. Reed, A. M., M. D., President of the American Medical Association (1900-1901); Gynecologist and Clinical Lecturer on Surgical Diseases of Women at the Cincinnati Hospital; Fellow of the American Association of Obstetricians and Gynecologists; Fellow of the British Gynecological Society, etc. Octavo, pp. 925. Illustrated by R. J. Hopkins. New York: D. Appleton & Co. 1901. [Price, $5.00.]

When some months ago the announcement was made that a new treatise on gynecology was soon to appear, edited and partly written by the distinguished president of the American Medical Association, it set that portion of the profession interested in that topic all agog; because, in part, of Dr. Reed's wide acquaintance with gynecologists in both hemispheres, and again, because of his resourcefulness as a clinician, his originality as a teacher, and his skilfulness at the operating table.

It is a pleasure to discover, after a careful examination of the book in question, that no disappointment lurks in its pages. It is essentially a text-book of gynecology, containing much that is original on that branch of medicine, while all of it is newly arranged, practically grouped and forcefully presented. It follows the fashion of the day in its encyclopedic form, but it differs from most books prepared in that way on account of the clever manner in which the editor has woven the warp and woof of his fabric; so cleverly, indeed, has this been done that the expert, unless he glanced at the list of contributors preceding the table of contents, would hardly dream that it was not a book by a single author.

The following-named writers, most of whom are widely known, appear in the list of contributors: J. W. Ballantyne, Edinburgh; J. H. Carstens, Detroit; Murdoch Cameron, Glasgow; Henry C. Coe,

PROFESSOR CHARLES A. L. REED, M. D.,

President American Medical Association, 1901,

Author-Editor of Reed's Gynecology.

New York; John G. Clark, Philadelphia; F. X. Dercum, Philadelphia; Walter B. Dorsett, St. Louis; L. H. Dunning, Indianapolis; Frank P. Foster, New York; Samuel G. Gant, New York; Hobart Amory Hare, Philadelphia, Malcolm L. Harris, Chicago; Maximilian Herzog, Chicago; R. J. Hopkins (artist) New York; Joseph Tabor Johnson, Washington; Wyatt G. Johnston, Montreal; Matthew D. Mann, Buffalo; Thomas Charles Martin, Cleveland; Lewis S. McMurtry, Louisville; Dan Millikin, Hamilton, O.; Henry P. Newman, Chicago; William Warren Potter, Buffalo; A. Ravogli, Cincinnati; Charles A. L. Reed, Cincinnati; Hunter Robb, Cleveland; James F. W. Ross, Toronto; A. W. Mayo Robson, Leeds; J. L. Rothrock, St. Paul; W. Japp Sinclair, Manchester; Horace J. Whitacre, and E. Gustave Zinke, Cincinnati.

One of the important features of this work, wherein, too, it differs from most of its kind, is the importance given to general or systemic treatment. This will give it a strong hold upon that large number of physicians who, not being specialists yet practice gynecology to a large extent and, withal, intelligently. The surgical part of the work, which, of course, constitutes its greater bulk, is exceptionally well handled; indeed, for the most part it is in advance of anything yet published on operative gynecology. Many of the methods described are new in technic. The descriptive text and the well-drawn illustrations make all such extremely clear, even to the inexperienced eye.

There are several subjects which do not usually appear in works of this kind, or at least, if they do, they are not elaborated by experts and specialists in the several topics. For instance, the relation of pelvic diseases and nervous affections is described by a neurologist, Dr. Dercum; neoplasms of the rectum are described by a proctologist, Dr. Gant; and the relation of diseases of the skin to gynecology are told by a dermatologist, Dr. Ravogli. Again, whatever relates to bacteriology, pathology, hygiene and other similar topics is written by men skilled in these several branches; hence, it often happens that several different authors appear as contributors to a single chapter. The advantage of this plan becomes apparent to every experienced gynecologist. It constitutes one of the unique features of this book, and the editor has so cleverly interwoven the lines of the various authors, often adding observations and comments of his own, that the whole literary and scientific fabric has become amalgamated into one homologous structure. It is doubtful if this has ever been as cleverly done in a work of poly-authorship.

An incisive part of this work is that relating to sepsis and antisepsis. It is very essential that the gynecological tyro shall have definite knowledge of the relations these two subjects bear to each other, and to gynecology in general. The editor has prepared what is said on this subject with great care and conciseness. Closely allied to it are the infections, and these are also discoursed upon by the editor, with the exception of that portion on tuberculosis, which is written by Dr. Whitacre, and another portion on infections of the uterus, written by Dr. McMurtry.

The mechanical make-up of the book deserves particular notice. In the first place, the simplicity of the title-page impresses the observer at once, because it is in such contrast to many of the grandiose airs that pervade the title pages of medical treatises nowadays. The illustrations by Mr. Hopkins, whether judged from the artistic or scientific viewpoint are of a superb character. They are for the most part original, more than nine-tenths never until now having appeared in gynecological literature. The legends descriptive of the figures have been extracted from the text, after the fashion prevailing in current fiction. We confess to a strong liking of this unique feature. A most comprehensive and well-prepared index closes this attractive, scientific and modern text-book of gynecology that will command the attention of every gynecologist in both hemispheres.

---

THE PATHOLOGY AND SURGICAL TREATMENT OF TUMORS. BY N. SENN, M. D., Ph. D., LL. D., Professor of Surgery, Rush Medical College in Affiliation with the University of Chicago; Professor of Surgery, Chicago Polyclinic; Attending Surgeon to Presbyterian Hospital; Surgeon-in-chief, St. Joseph's Hospital, Chicago. 8vo, pp. 718. Second edition, revised. Illustrated by 478 engravings, and 12 full-page plates in colors. Philadelphia: W. B. Saunders & Co. 1900. (Price cloth, $5 00, net; half-Morocco, $6.00 net.)

That within a few years a second edition of Dr. Senn's large book on tumors has been called for, speaks well for the reception accorded the first. The first edition has been so long before the medical public that they have had ample opportunity to become acquainted with its general characteristics. It was essentially a general handbook of the subject in its pathological and clinical aspects, and formed a very useful work in that its readers could get a comprehensive grasp, both of the nature of various growths and the best methods by which to treat them. It evinced a large amount of research, although it had about it very little which was distinctly original. Dr. Senn is essentially an encyclopedist rather than a great original student. When now we come to consider the second edition, we find that it contains all the good characteristics which marked the first, and that considerable has been added to it. When, however, one takes up the book with the endeavor to acquire the very latest information about some of the disputed pathology of tumors, he is necessarily disappointed, since the book contains but little of that which one would expect to find in this direction.

First of all, the index is very inadequate and disappointing, and one must search through many pages before he decides whether a given subject is treated or not. In the second place, authors and authorities are frequently mentioned and ample credit given in this way, but should one wish to consult their articles he finds few, if any references to volume and page, and must hunt these up from some other source.

One would certainly have a right to turn to this book for a résumé of Wilm's papers on the general subject of teratoma; and also for information concerning that particular form of adrenal tumor which has now been amply described, first, by Grawitz, and in this country by Kelly and others, known as hypernephroma. The word does not appear in the index, nor is there any mention of teratomata of the kidney. So, also, in the case of that other mixed tumor of the kidney which has been amply and repeatedly described, and which has gone by so many names, among them, cystadenoma, adenosarcoma, etc., essentially of congenital origin. The writer has been able to find no description of it whatever in this new and revised edition. Again, in the index, the word endothelioma does not appear, and one is compelled to hunt through the text before he finds any mention of the subject, and even then he finds a very inadequate description of it, although it has been most carefully written up by Volkmann and by others. Still more is this true of perithelioma, which appears neither in the index nor text. The same also of cylindroma, which certainly deserves mention, whether it be regarded according to the old views of Bilroth, or under the modern classification as a sub-variety of sarcoma.

Under the heading of sarcoma, no distinct nor adequate description of osteosarcoma is given, while that given of angiosarcoma is inadequate and very misleading. The author has failed to make it clear that there is a great difference between sarcoma of bone and osteosarcoma, the former referring to a tumor proceeding from the periosteum or medulla, while the latter is a tumor originating in the original stroma or texture of the tissue which shall later become bone.

There is a short description of deciduoma malignum, although it is not called by the name now generally given to it of chorion epithelioma. Moreover, a distinct error is here made in that it is spoken of as being occasionally of sarcomatous character, whereas the studies of Marchand have placed the epithelial nature of all these tumors beyond a doubt. The author, in his consideration, has evidently followed Sänger, neglecting the later and much more accurate studies, for now nobody but a gynecologist would hold to its sarcomatous nature.

But it is ungracious to continually find fault with a book, only one must necessarily have a feeling that this is a work to which he has a right to turn for the very latest information, in which feeling he will receive a very distinct disappointment. It would have been much better for the profession had this book been retained a time longer in order that all this new matter might have been inserted. Nevertheless, it is a great help to the general practitioner and one may find but little fault with that which it does contain, while regretting that which it does not. The author is evidently no friend of the parasitic nature of malignant growths, and by no means does adequate justice to the work which has been done to establish this hypothesis. In fact, it would almost seem as if he were like Holmes's theologians who close their nicitating membranes, or third eyelids, toward that which they do not care to see. Many of the illustrations are good, especially the newer ones, while some of them are quite shabby, and by no means up to the standard of such a representative work as this is supposed to be. Let us hope that the author will before long prepare a third edition, in which one may look for at least an epitomé of the whole subject in its modern aspect and experience no disappointment.

CANCER OF THE UTERUS. Its Pathology, Symptomatology, Diagnosis and Treatment. Also the Pathology of Diseases of the Endometrium. By THOMAS STEPHEN CULLEN, M. B. (Toronto), Associate Professor of Gynecology in the Johns Hopkins University. Imperial 8vo, pp. 709, with 11 lithographic plates and over 300 colored and black illustrations in the text, by Max Brodel and Hermann Becker. New York: D. Appleton & Co. 1901. [Prices, cloth, $7.50; half morocco, $8.50.] By subscription only.

Without doubt cancer of the uterus, all things considered, is the most terrible malady that can afflict woman. The object of this volume is to aid the physician, and especially the family doctor, in making an early diagnosis of the dread malady. To this end the author has pointed out not only the early signs of carcinoma, but he also records in detail such diseases as may be mistaken for or confounded with cancer. His chapter on the anatomy of the uterus is one of the most accurate that has been published, and the illustrations are so correct and clear as to charm the reader at the very beginning of the book.

After devoting a chapter to the removal and examination of uterine tissues for diagnostic purposes, the author proceeds to the consideration of the squamous-cell carcinoma of the cervix, which occupies about 240 pages of the book. It is beautifully illustrated with plates, engravings and half-tones, showing every stage in the pathology of this, the commonest form of cancer of the uterus. One of the worst features to deal with in this form of the disease is, the fact that the new growth often proceeds to a stage of considerable advancement without attracting the attention of the victim. Too often has the family doctor treated with indifference the first interview with his patient who may incidentally mention some slight uterine symptom. If he will read these pages with care, he will become convinced that it is of the first importance to carefully investigate every case that presents itself to him with the slightest suspicion.

It becomes the emphatic duty of the physician particularly to inquire into any departure from the normal cessation of the menopause, this being a time when there is great liability to the invasion of cancer. The carefully recorded clinical histories that are so copiously reported do much to aid in coming to a clear understanding of this subject.

The author allots about 300 pages of the text next following to the consideration of adenocarcinoma of the cervix and body of the uterus. This, like the preceding section of the book, is illustrated with admirable accuracy of detail as well as great profuseness, here and there color being used to give strength to the contrast in pathological tissue. Here, too, are to be found clinical histories almost without number, amplifying and illustrating the subject in great detail.

In the remaining chapters treatment and rare conditions are handled, as well as complications, such as pregnancy, together with prognosis and etiology; also, some inoculation experiments are given, bearing upon the general question of the nature and cause of the disease.

The book is printed on heavy plate paper with clear-face type and, with its excellent illustrations, presents as beautiful a specimen of the book-making art as one will see in many a day. Moreover, it is a wonderful book. It is a royal octavo volume of 693 pages, entirely devoted to the consideration of one disease, in its attacks upon one organ of the body in one half of the human race. It is one of the most useful books that has lately issued from the press and ought to do much toward restraining the ravages of the terrible disease with which it deals.

A Text-Book on Practical Obstetrics. By Egbert H. Grandin, M. D., Gynecologist to the Columbus Hospital; Consulting Gynecologist to the French Hospital, etc. With the collaboration of George W. Jarman, M. D, Gynecologist to the Cancer Hospital. Third edition, revised and enlarged. Illustrated with 52 full-page photographic plates and 105 illustrations in the text. Pages xiv.—511. Philadelphia: F. A. Davis Company. [Price, cloth, $4.00 net; sheep, $4.75 net.]

The first edition of this book appeared in 1894. In it an attempt was made to revolutionise the methods of instruction in obstetrics. Despite its defects and the many obstacles it met, it was partially successful in its mission.

This third edition has been very much enlarged beyond the limits of the original treatise, and some additions have been made, notably a chapter on the anatomy of the female organs of generation, and one on embryology. Some changes, too, have been made in the arrangement of the illustrations, by reducing the size of a number and grouping them, which adds to the homogeneity of the volume.

While there are still some defects, and a few errors in the book, it is yet a valuable addition to the obstetric department of the library, and will easily find a place there. Both teachers and practical obstetricians among the general profession will find it a valuable aid.

---

International Clinics. A Quarterly of Clinical Lectures and Especially Prepared Articles on Medicine, Neurology, Surgery, Therapeutics, Obstetrics, Pediatrics, Pathology, Dermatology, Diseases of the Eye, Ear, Nose and Throat, etc. Edited by Henry W. Cattell, A. M., M. D., of Philadelphia, with the collaboration of Jonn B. Murphy, M. D., of Chicago; Alexander D. Blackader, M. D., of Montreal; H. C. Wood, M. D., of Philadelphia; T. M. Rotch, M. D., of Boston; E. Landolt, M. D., of Paris; Thomas G. Morton, M. D., and Charles H. Reed, M. D., of Philadelphia; J W. Ballantyne, M. D., of Edinburgh, and John Harold, M. D., of London. Volume IV., Tenth series; and Volume I., Eleventh series. 1901. Philadelphia; J. B. Lippincott Company.

I. The first of the volumes above noted contains lectures on therapeutics, genito-urinary diseases, medicine, neurology, surgery, pathology and laboratory methods, together with a monograph by Henry W. Cattell on the eitology and morbid anatomy of various diseases. The volume opens with an interesting article on the United States Pharmacopeia by Horatio C. Wood, and the department of therapeutics also contains an article of value on Mosquitoes and the Prophylaxis of Malaria by Professor Grassi.

In the symposium on genito-urinary diseases, James Pedersen lectures on the treatment of urethritis in the male. This is a most instructive contribution to the therapeutics of this disease. John B. Deaver contributes a lecture on several surgical diseases which constitutes the entire contribution to the department of surgery in the book. It is made up of abstracts from the public Saturday clinical lectures delivered by the author at the German Hospital, Philadelphia. The material for these lectures was secured from reports furnished by several young physicians in competition for a prize offered by Dr. Deaver for the best accounts of his clinics. This is a most commendable method to secure accuracy and good material for publication and this series will be continued in future issues of this work.

II. Volume I. of the eleventh series contains lectures on therapeutics, medicine, neurology, surgery, obstetrics and gynecology, diseases of the eye, laboratory methods, together with a review of the progress of medicine during the year 1900, prepared by N. J. Blackwood. Dr. Deaver continues the abstracts of his clinics referred to in our notice of the previous volume, this time, however, in the department of gynecology. There are a number of other contributors of note to this book and it is a volume of interest. Indeed, these two are among the best numbers of the clinics yet issued.

## Figures and Tables

**Figure f1:**